# Weight gain and physical inactivity during the COVID-19 pandemic

**DOI:** 10.26633/RPSP.2021.136

**Published:** 2021-10-18

**Authors:** Soraya Bruno Gutierrez, Karla Orihuela Quispe

**Affiliations:** 1 Universidad Privada San Juan Bautista Lima Perú Universidad Privada San Juan Bautista, Lima, Perú.

To the editor:

In the article by Sánchez Gómez et al. (1) authors state that “microeconomic studies for the treatment of obesity have increased in recent years”. We must bear in mind, however, that this does not happen worldwide. The country with most evidence about this topic is the United States, since 40% of its population is obese. In other countries, however, more research in this field is needed, and this is a cause for concern as obesity is a disease whose treatment incurs avoidable costs that have increased during the pandemic.

COVID-19 infection is transmitted from person to person, so one of the measures taken to reduce its rapid spread, as well as the infection rate and associated mortality, is the social isolation applied in most countries of the world. As a result, weight control, physical inactivity and changes in eating habits have become worrying issues. Various studies have shown that small changes in body weight in relatively short periods can become permanent and lead to substantial weight gain over time (2).

These changes can be associated with two important aspects. First, staying at home, which includes working remotely, studying, or spending many hours per day in front of a computer with little outdoor physical activity. Second, the storage of food at home due to the existing restrictions to go outside to purchase food. In addition, the interruption of the work routine caused by quarantine could result in boredom, which in turn might be associated with an increased caloric intake. The stress with which most people are currently living might also cause overeating, mainly of carbohydrate-rich foods, which carries a higher risk of developing obesity, which has been shown to increase the risk of more serious complications of COVID-19 infection (3).

Studies conducted in different countries focused on how eating habits, physical activity and weight gain were affected by the COVID-19 pandemic. In Italy, a study was conducted with 3 533 people aged between 12 and 86; the majority of participants (46.1%) had not changed their eating habits while 37% felt that these have worsened and 48.6% perceived a weight increase (3). In Poland, 1 097 people were surveyed, with a majority of women which had a normal body mass index. From them, 51.8% admitted eating snacks between meals more often and 43.5% reported eating more during quarantine periods. In this study, 29.9% reported weight gain, and weight change was correlated with body mass index and age (4). In a study from the United Arab Emirates with 1 012 subjects, an increase in the percentage of participants consuming five or more meals per day was shown, from 2.1% before the pandemic to 7% during it. With regard to physical activity, prior to the pandemic 32.1% did no physical exercise, a percentage that increased to 36.5% during quarantine periods; 40.3% also noticed an increase in body weight. These results indicate that in the UAE the study population experienced negative lifestyle changes, unbalanced food choices, and a reduction in physical activity during the COVID-19 pandemic (5).

It is evident that obesity has been increasing, and despite prevention being an effective approach there has always been more emphasis on treatment, including diet changes and weight loss surgery. As obesity is accompanied by other more serious pathologies, which produce even more costs for health systems, countries should investigate more about the economic impact of obesity to understand the shortcomings of the health systems and together with nutritionists strengthen the population’s knowledge about actions to prevent obesity.

## Disclaimer.

Authors hold sole responsibility for the views expressed in the manuscript, which may not necessarily reflect the opinion or policy of the RPSP/PAJPH or the Pan American Health Organization (PAHO).
